# It’s Time for Innovation in the Health Insurance Portability and Accountability Act (HIPAA)

**DOI:** 10.2196/medinform.6372

**Published:** 2016-11-02

**Authors:** Karen Colorafi, Bryan Bailey

**Affiliations:** ^1^ College of Nursing Washington State University Spokane, WA United States; ^2^ Millligan Lawless Phoenix, AZ United States

**Keywords:** innovation, HIPAA, electronic health record demonstration

## Abstract

Whether it is the result of a tragic news story, a thoughtful commentary, or a segment on the entertainment networks, patient privacy rights are never far from the top of our minds. The Privacy and Security Rules contained in the Health Insurance Portability and Accountability Act of 1996 (HIPAA) represent a concerted effort to protect the privacy and security of the volumes of patient data generated by the health care system. However, the last twenty years has seen innovations and advancements in health information technology that were unimaginable at that time. It is time for innovation to the Privacy and Security Rules. We offer a common and relatable scenario as proof that certain Privacy and Security Rules can tie the hands of educators and innovators and need to be transformed.

## Introduction

Recently we came across an art exhibit hosted by a prestigious American school in which a portable printer was set to download the messages sent through a hospital’s digital pager system. We understand the artist stumbled upon the messages innocently one day while scanning various radio frequencies. The realization that pager data was so easily accessible prompted the artist to create the unique installation. This bold and creative act calls our attention to the abundance of intricate technology in our health care system, the lack of intention to the unintended consequences of its use, and the need we have to deploy technology safely. In other words, there is an innovation gap in play.

Weiss and Legrand (2011) define the innovation gap as the difference between the stated importance of innovation and the actual results achieved in an organization [1]. In its day, the Health Insurance Portability and Accountability Act of 1996 (HIPAA) represented a significant advance: society’s commitment to the protection of patient data, defending their rights by keeping sensitive health care information private and secure. Over a decade later, the Health Information Technology for Economic and Clinical Health (HITECH) Act [2] included in the American Recovery and Reinvestment Act (ARRA) stimulus legislation (2009) acknowledged some of the technological enhancements associated with the science of health care delivery and increased the penalties associated with violating the Act in a collective effort to promote the proper guardianship of health care data (Department of Health and Human Services, DHHS) [3]. The Security Rule was created with unusual foresight as a set of flexible requirements that could change and adapt with innovation.

Yet every week, the headlines online and in the papers discuss significant HIPAA infractions. The US Office for Civil Rights maintains a website dedicated to the public reporting of breaches affecting 500 or more individuals [4]. Online bloggers have publicly questioned whether details leaked to the press about the circumstances surrounding the recent death of the artist Prince constituted a HIPAA violation [5], illustrating the heightened anxiety the general public feels about the ability of the health care profession to adequately protect the privacy and security of health care data. The scholarly literature continues to report that concerns about data breaches is a chief concern of patients, ultimately affecting the trust a patient places in a provider and in a health care facility [6]. We listen to stories from our friends and patients about the battles they have mounted to gain access to their own health care data.

We wrestle within our own organizations to make sense of HIPAA and to deploy its requirements responsibly while rolling out the next generation of health information technology (HIT), like real-time clinical dashboards and apps. Some have argued the iniquitousness of a rule that applies to health care apps but not consumer apps, even when they contain similar information [7-9]. We struggle to train a new generation of health care providers on electronic health record (EHR) systems and we refuse to share data with researchers out of fear of violating the rules. In short, it seems at times that our use of the Privacy and Security Rules has not adapted or supported the achievements and demands of health care.

We propose that health care leaders consider the significance of the innovation gap by deliberating a common scenario, one encountered by the authors on a regular basis: the EHR demonstration. Leaders in health care facilities who are justifiably proud of their EHR system are often approached by colleagues, educators, and vendor prospects to give demonstrations. Demonstrations are conducted for a variety of purposes: to show a colleague something that is especially fantastic or problematic with a particular system, to train health care providers, clinicians, or support staff, or to close a big sale. While the opportunity to showcase a beautiful system seems like a helpful thing to do – a professional courtesy of sorts – the facility (“covered entity”) ought to carefully consider its responsibilities under the Act before agreeing to provide a demonstration.

Recently, one of the authors attended three different EHR demonstrations alongside a group of health care administration graduate students. Each of the demonstrations was given in a live production database and two out of the three used real patient encounters to demonstrate various scheduling, registration, billing, and clinical documentation scenarios. One student whose wife was a patient in one of the practices spent the entire session overwhelmed with anxiety that the next record revealed would be one with which he was intimately familiar. This viewpoint provides health care leaders with a short review of HIPAA essentials, offers a compelling scenario suggesting the need for innovation, and provides suggested approaches to protecting patient privacy, working within the current confines of the HIPAA Privacy and Security Rules.

## What is Protected Health Information?

Protected health information (PHI) includes all individually identifiable health information held or transmitted by a covered entity (or its business associates) in any form. Individually, identifiable health information is that which is created or received by a health care provider, health plan, employer, or health care clearinghouse which (1) relates to the past, present, or future physical or mental health or condition of an individual, the provision of health care to an individual, or the past, present, or future payment for the provision of health care to an individual; and (2) either identifies the individual or can be used to identify the individual. Electronic protected health information (e-PHI) is PHI that is maintained or transmitted in an electronic media, such as an EHR or practice management system and is afforded the same protections.

## Why Do I Have to Protect It?

The Privacy Rule prohibits covered entities from using and disclosing PHI (including e-PHI), except as permitted or required by the Rule. The Security Rule requires covered entities to maintain reasonable and appropriate administrative, technical, and physical safeguards to protect e-PHI. For example, a covered entity must ensure the confidentiality of, anticipate threats to, and protect against impermissible uses and disclosures of e-PHI that resides in an EHR or practice management system by using safeguards such as complex and changing passwords, firewalls, and locking the server room. Failing to comply with the Privacy or Security Rule may result in civil monetary and criminal penalties. In addition, violations of the Privacy Rule may require written notifications of the impermissible use or disclosure to the affected individual(s), the Office for Civil Rights, and the media.

## When Can Protected Health Information Be Used or Disclosed?

Generally speaking, the Privacy Rule prohibits covered entities from using or disclosing an individual’s PHI without first obtaining the individual’s prior written authorization. However, there are a number of exceptions to this Rule (Textbox 1).

**Exceptions to the Privacy Rule.**

1. Giving information to the individual.

2. For treatment, payment, and health care operations (see [10] for a quick definition of these terms).

3. To persons involved in the individual’s care after providing the individual with an opportunity to verbally agree or object, except in emergencies (eg, using the individual’s name in a facility directory, paying a spouse’s bill, and picking up a prescription for a family member).

4. Incidental disclosures of PHI resulting from a permitted use or disclosure (eg, a person glimpses another patient’s name on a sign-in sheet).

5. For certain public interest purposes, such as disclosures that are required by law (eg, communicable diseases or child abuse).

## What Can Be Disclosed?

At best, it is unclear whether a covered entity can disclose PHI during a demonstration. If a health care facility was under investigation for a violation, you might retrospectively argue that the Privacy Rule’s definition of “health care operations” includes “training programs in which students, trainees, or practitioners in areas of health care learn under supervision to practice or improve their skills as health care providers” and “training of non-health care professionals”. After all, a reasonable person may question how we plan to adequately train a new generation of programmers, information technology professionals, data scientists, business administration, and clinical students without acquainting them with one of health care’s most powerful tools. However, we would *not* prospectively advise a covered entity to disclose PHI during a demo based on this argument. Even if the disclosure is permitted, the covered entity still must comply with the Privacy Rule’s minimum necessary and reasonable safeguard requirements, which means the covered entity must have reasonable safeguards in place to ensure it only discloses the minimum PHI necessary for the demo. This is easier said than done. For these reasons, practices are safer by not disclosing any PHI during a demo. In addition, since there is a risk of improper and incidental disclosures of PHI while the demo participants are in your office, you must ensure that safeguards are in place to minimize these risks.

## What Should I Do?

Tips that can help you prepare for a satisfying EHR demonstration while fulfilling your obligations under the Privacy and Security Rules are shown in Textbox 2.

It is important to remember that innovation does not simply happen once. A learning organization will revisit their policies and procedures related to the protection of data at least annually, or when a change in infrastructure demands (another requirement of the Act). Furthermore, we ought to consider that an Act that was innovative in 1996 may no longer solve the problems it was created to address, partly because the nature of the problem has changed. Academia has a desperate need to train students on the optimal use of EHR and practice management systems, which are commonplace across the country and represent the new standard of care. Health care businesses have an urgent need to partner with professionals and scholars who can analyze and make sense of their own EHR data. Industry could innovate and invent solutions to pressing and costly problems with adequate access to information. However, health professions training, big data, pharmacogenetics, and the re-selling of health care datasets are issues scantly addressed by the Act. We are well served to remember that innovation is best thought of as a process, not an outcome, that occurs within social environments that are dynamic and constantly changing [11]. We posit that health care needs innovation in the Privacy and Security rules to address the complexity that is inherent within the system in which we work and seek care.

**Tips to help you prepare for a satisfying EHR demonstration.**

- Develop a policy and procedure for your HIPAA Privacy and Security set to explain the rules governing demonstrations of your EHR or practice management systems.
- Educate staff on the Privacy and Security Rules and your privacy and security policies and procedures (eg, be clear about what constitutes PHI, such as names on schedules).
- Always demo out of a test, build, or train (non-production) database.
- Ensure that the demo database does not contain actual PHI (sometimes configuration databases are back-loaded with real patient data from the live system).
- If you do not have a unique demo database:
  - Make sure there is a unique demo user login to your production database that does not have access to live patient data (eg, tasks, documents, and labs to review) and instead, demo test patients (eg, Donald Duck, James Cerner, and Abbey Allscripts).
  - Consider preparing a demo using screen shots (PHI redacted) on PowerPoint slides instead of using your production EHR. This is especially effective with “live” technologies such as telemedicine systems or state-run drug database inquiries.
- If appropriate to your situation, ensure your guests have signed a business associate agreement.
- Keep a log of dates and times when demos were provided and the names of attendees.
- Ask attendees to put mobile phones and tablets (eg, devices with cameras) in a basket before the demo begins and give them back when the demo is complete.

## Abbreviations

EHR: electronic health record
e-PHI: electronic protected health information
HIPAA: Health Insurance Portability and Accountability Act
PHI: protected health information
